# 2184. Clinical and Molecular Epidemiology of Carbapenem-Resistant Gram-Negative Bacilli in a Los Angeles Tertiary Medical Center

**DOI:** 10.1093/ofid/ofad500.1806

**Published:** 2023-11-27

**Authors:** John Michael Sanchez, Deisy Contreras, Margie Ann Morgan

**Affiliations:** Cedars-Sinai Medical Center, Los Angeles, California; Cedars-Sinai Medical Center, Los Angeles, California; Cedars-Sinai Medical Center, Los Angeles, California

## Abstract

**Background:**

Multidrug-resistant gram-negative bacilli are a significant public health threat, associated with 2.2 million deaths worldwide. Of particular importance is the rise of carbapenem-resistant gram-negative bacilli (CRGNB) given the limited options for treatment. The 30-day mortality associated with carbapenem-resistant *Pseudomonas aeruginosa*, carbapenem-resistant Enterobacterales, and carbapenem-resistant *Acinetobacter baumannii* ranges from 16 to 24%. In recognition of the substantial danger of these organisms, the World Health Organization has designated three CRGNB as Critical Priority pathogens.

**Methods:**

Based on culture data and antimicrobial susceptibility testing, patients with CRGNB at Cedars-Sinai Medical Center from December 2022 to April 2023 were identified. Clinical data including patient demographics, prior carbapenem use, isolation of additional antibiotic-resistant organisms, subsequent antibiotic therapy, and mortality were compared among CRGNB.

**Results:**

Of over 65 isolates, the largest proportion of isolates were cultured from pulmonary sources (44%), followed by urine (34%), skin (12%), blood (6%), and abdominal sources (4%). The most prevalent CRGNB isolated were *Pseudomonas aeruginosa* (65%), *Escherichia coli* (13%), and *Klebsiella pneumoniae* (7%). A majority of patients with CRGNB had prior documented carbapenem use (74%) and 71% of CRGNB were associated with at least one other antibiotic resistant organism. Cefiderocol was used to treat 12% of cases. However, 24% of CRGNB were not treated with antibiotics, often with the rationale that the isolate was likely a colonizing strain. The mortality rate among patients with CRGNB during our observation period was 18%.

**Conclusion:**

CRGNB are emerging pathogens associated with prior carbapenem use and result in significant mortality. Subsets of organisms from our dataset will be further studied for relatedness using Fourier-Transform infrared spectroscopy and whole genome sequencing.

**Disclosures:**

**All Authors**: No reported disclosures

